# Vibration frequency analysis of three-layered cylinder shaped shell with effect of FGM central layer thickness

**DOI:** 10.1038/s41598-018-38122-0

**Published:** 2019-02-07

**Authors:** Madiha Ghamkhar, Muhammad Nawaz Naeem, Muhammad Imran, Muhammad Kamran, Constantinos Soutis

**Affiliations:** 1 Department of Mathematics, Government College University Faisalabad, Faisalabad, Pakistan; 2 Department of Mathematics, COMSATS University Islamabad, Wah Campus, Islamabad, Pakistan; 3 Aerospace Research Institute and Northwest Composites Centre, The University of Manchester, Manchester, UK

## Abstract

In this research, vibration frequency analysis of three layered functionally graded material (FGM) cylinder-shaped shell is studied with FGM central layer and the internal and external layers are of homogenous material. Strain and curvature-displacement relations are taken from Sander’s shell theory. The shell frequency equation is obtained by employing the Rayleigh Ritz method. Influence on natural frequencies (NFs) is observed for various thickness of the middle layer. The characteristics beam functions are used to estimate the dependence of axial modal. Results are obtained for thickness to radius ratios and length to radius ratios for different edge conditions. The validity of this method is checked for numerous results in the open literature.

## Introduction

Vibration of FGM cylindrical shell is a widely studied area of research in theoretical and applied mechanics. Among a large number of studies on vibrations of cylindrical shells (CS) we cite a few. Arnold and War-burton^1,2^ is executed some influential work on shell frequency analysis. Shell vibration analysis carried out by employing different numerical techniques like Galerkin method, Rayleigh Ritz method, different quadrature method and finite difference method. These shells are fabricated by isotropic, laminated and multi-layered materials. Functionally graded materials have been developed by applying powder technology. Functionally graded materials are utilized for various objectives because of their proper material distribution in their fabrication. They are mostly used for high pressure and heat dominant surroundings. Sharma *et al*.^3^ scrutinized behaviour of vibrations for cylinder-shaped shells by employing the Rayleigh Ritz technique for clamped-free boundary conditions. Loy *et al*.^4^ analysed the fundamental frequencies of circular shaped shells by a generalized differential quadrature method (DQM). Further Loy *et al*.^5^ investigated the vibrations of functionally graded (FG) cylindrical shells fabricated by stainless steel and nickel. They showed the effects of formations of essential constituents on the frequencies. Moreover, Pardhan *et al*.^6^ explored vibration behaviour of FG cylindrical shells fabricated by stainless steel and zirconia for different edge conditions. Zhang *et al*.^7^ scrutinized free vibrations of cylindrical shells for different edge conditions by employing a local adaptive DQM. Naeem *et al*.^8^ employed a generalized DQM for the functionally graded material cylindrical shells to investigate vibration behaviour. Pellicano^9^ showed the response of an isotropic cylindrical shell for linear and non-linear vibrations by employing analytical experiment method. Vibration study of FG cylindrical shells has been done by Iqbal *et al*.^10^ and the shell governing motion equations were solved by using wave propagation technique. This technique was exceptionally helpful for vibration analysis. Axial modal dependence was estimated with help of beam functions in exponential form. Li *et al*.^11^ determined free vibration analysis of three layered cylindrical shells with FG material central layer. Flugge’s shell theory was used by them. Vel^12^ observed free and forced vibration of cylinder-shaped shell by using the elasticity solution technique for simply - supported conditions at both ends. Lam *et al*.^13^ showed the frequency vibration behaviour of multi layered FGM cylindrical shells for different edge conditions. Arshad *et al*.^14,15^ studied the FGM cylindrical shell for vibration frequency analysis with simply - supported end point conditions under different volume fraction laws. They used Love’s shell theory. Rayleigh Ritz technique was employed by them to solve the problem. Further he investigated vibration characteristics of FGM cylindrical shell with the effect of different edge conditions for exponential volume fraction law. Shah *et al*.^16^ analysed vibrations of NFs for fluid filled and empty CS constructed by elastic foundation. Naeem *et al*.^17^ explored the vibration behaviour of three layered functionally graded material cylindrical shell for different edge conditions. The internal and external layers were fabricated by FG materials whereas the central layer was of isotropic material. They used the Love’s thin shell theory. Arshad *et al*.^18^ examined the vibrations of natural frequencies of bi-layered cylinder-shaped shell. One layer was fabricated by isotropic material and the other was of functionally graded material. Rayleigh Ritz technique was utilized. Shah *et al*.^19^ scrutinized the vibration behaviour of three layered FGM CS constructed by Winkler and Pasternak basis. They used wave propagation approach for the solution of the model.

Ahmad and Naeem^20^ investigated vibrations of rotating cylindrical shells composed of FG materials. Natural frequencies of cylindrical shell were studied with effects of volume fraction law and different ratios.

## Theoretical Consideration

**Figure 1:**
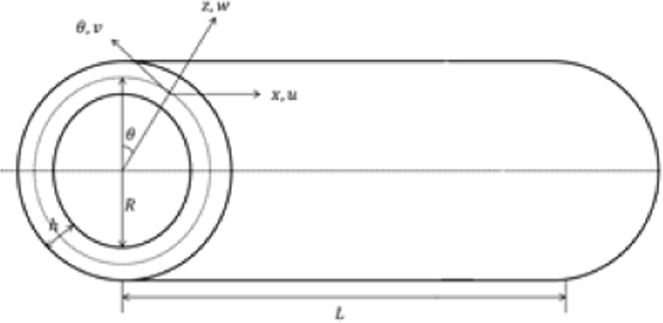
Geometry of three layered FGM CS.

Consider a cylinder-shaped shell of radius *R*, thickness *h* and length *L* as shown in Fig. 1. An orthogonal coordinate system (*x*, *θ*, *z*) is fixed at the middle surface of the cylindrical shell, where *x*, *θ* and *z* lie in the axial, circumferential and radial directions of the shell, and (*u*, *v*, *w*) are the displacements of the shell in *x*, *θ* and *z* directions respectively.

The strain energy for a CS is represented by $\Im$ and is written as

1 $$\Im =\frac{1}{2}{\int }_{0}^{L}{\int }_{0}^{2\pi }\{\mathop{{\rm{K}}}\limits_{\mbox{'}}\}^{\prime} [S]\{\mathop{{\rm{K}}}\limits_{\mbox{'}}\}Rd\theta dx,$$

where

2 $$\{\mathop{{\rm{K}}}\limits_{\mbox{'}}\}^{\prime} =\{{\varepsilon }_{1},\,{\varepsilon }_{2},\,\gamma ,\,{K}_{1},\,{K}_{2},\,2\tau \},$$

where *ε*_1_, *ε*_2_, *γ* and *K*_1_, *K*_2_, *τ* represent the strains and curvatures reference surface relations respectively. Prime (′) denotes the transpose of a matrix. These relations are taken from Sanders’ shell theory and written as:

3 $$\{{\varepsilon }_{1},{\varepsilon }_{2},\gamma \}=\{\frac{\partial u}{\partial x},\frac{1}{R}(\frac{\partial v}{\partial \theta }+w),(\frac{\partial v}{\partial x}+\frac{1}{R}\frac{\partial u}{\partial \theta })\},$$

4 $$\{{K}_{1},{K}_{2},\tau \}=\{\,-\frac{{\partial }^{2}w}{\partial {x}^{2}},-\frac{1}{{R}^{2}}(\frac{{\partial }^{2}w}{\partial {\theta }^{2}}-\frac{\partial v}{\partial \theta }),-\,\frac{2}{R}(\frac{{\partial }^{2}w}{\partial x\partial \theta }-\frac{3}{4}\frac{\partial v}{\partial x}+\frac{1}{4R}\frac{\partial u}{\partial \theta })\},$$

and [*S*] is defined as

5 $$[S]=[\begin{array}{llllll}{a}_{11} & {a}_{12} & 0 & {b}_{11} & {b}_{12} & 0\\ {a}_{12} & {a}_{22} & 0 & {b}_{12} & {b}_{22} & 0\\ 0 & 0 & {a}_{66} & 0 & 0 & {b}_{66}\\ {b}_{11} & {b}_{12} & 0 & {d}_{11} & {d}_{12} & 0\\ {b}_{12} & {b}_{22} & 0 & {d}_{12} & {d}_{22} & 0\\ 0 & 0 & {b}_{66} & 0 & 0 & {d}_{66}\end{array}],$$

where *a*_*ij*_ denote the extensional stiffness, *b*_*ij*_ the coupling stiffness and *d*_*ij*_ the bending stiffness. (*i, j* = *1*, *2* and 6). They are defined as:

6 $$\{{a}_{ij},{b}_{ij},{d}_{ij}\}={\int }_{-\frac{h}{2}}^{\frac{h}{2}}{{,}\kern-0.4em \rm{O}}_{ij}\{\mathrm{1,}\,z,\,{z}^{2}\}dz\mathrm{.}$$

For isotropic materials ${{,}\kern-0.4em \rm{O}}_{ij}$ is the reduced stiffness stated as Loy *et al*.^5^

7 $${{,}\kern-0.4em \rm{O}}_{11}={{,}\kern-0.4em \rm{O}}_{22}=E{\mathrm{{\rm{{-}\kern-0.5em \lambda}}}^{2})}^{-1},\,{{,}\kern-0.4em \rm{O}}_{12}={{\rm{{-}\kern-0.5em \lambda}}}E{\mathrm{{\rm{{-}\kern-0.5em \lambda}}}^{2})}^{-1},\,{{,}\kern-0.4em \rm{O}}_{66}=E{\mathrm{(2(1}+{{\rm{{-}\kern-0.5em \lambda}}}))}^{-1}.$$

Here Young’s modulus represented by *E* and ${{\rm{{-}\kern-0.5em \lambda}}}$ denotes the Poisson ratio. The *b*_*ij*_ coupling stiffness turn to zero for homogenous CS and ≠0 for FGM cylindrical shells and values of *b*_*ij*_ depend on the material distribution. Also *b*_*ij*_ become negative and positive due to irregularity of material properties at the mid plan. ${{,}\kern-0.4em \rm{O}}_{ij}$ depend on physical properties of FG materials.

With the help of expression (2) and (5), $\Im$ is written as:

8 $$\begin{array}{rcl}\Im  & = & \frac{1}{2}{\int }_{0}^{L}{\int }_{0}^{2\pi }\{{a}_{11}{{\varepsilon }_{1}}^{2}+{a}_{22}{{\varepsilon }_{2}}^{2}+2{a}_{12}{\varepsilon }_{1}{\varepsilon }_{2}+{a}_{66}{\gamma }^{2}+2{b}_{11}{\varepsilon }_{1}{K}_{1}\\  &  & +\,2{b}_{12}{\varepsilon }_{1}{K}_{2}+2{b}_{12}{\varepsilon }_{2}{K}_{1}+2{b}_{22}{\varepsilon }_{2}{K}_{2}+4{b}_{66}\gamma \tau \\  &  & +\,{d}_{11}{{K}_{1}}^{2}+{d}_{22}{{K}_{2}}^{2}+2{d}_{12}{K}_{1}{K}_{2}+4{d}_{66}{\tau }^{2}\}\,Rd\theta dx\mathrm{.}\end{array}$$

By putting these expressions (3) and (4) in the expression (8) then $\Im$ attains the following form:

9 $$\Im =\frac{R}{2}{\int }_{0}^{2\pi }{\int }_{0}^{L}[{a}_{11}{(\frac{\partial u}{\partial x})}^{2}+\frac{{a}_{22}}{{R}^{2}}{(\frac{\partial v}{\partial \theta }+w)}^{2}+\frac{2{a}_{12}}{R}\frac{\partial u}{\partial x}{(\frac{\partial v}{\partial \theta }+w)}^{2}+{a}_{66}{(\frac{\partial v}{\partial x}+\frac{1}{R}\frac{\partial u}{\partial \theta })}^{2}-{b}_{11}(\frac{\partial u}{\partial x})(\frac{{\partial }^{2}w}{\partial {x}^{2}})-\frac{2{b}_{12}}{{R}^{2}}(\frac{\partial u}{\partial x})(\frac{{\partial }^{2}w}{\partial {\theta }^{2}}-\frac{\partial v}{\partial \theta })-\frac{2{b}_{12}}{R}(\frac{\partial v}{\partial \theta }+w)(\frac{{\partial }^{2}w}{\partial {x}^{2}})-\frac{2{b}_{22}}{{R}^{3}}(\frac{\partial v}{\partial \theta }+w)(\frac{{\partial }^{2}w}{\partial {\theta }^{2}}-\frac{\partial v}{\partial \theta })-\frac{4{b}_{66}}{R}(\frac{\partial v}{\partial x}+\frac{1}{R}\frac{\partial u}{\partial \theta })(\frac{{\partial }^{2}w}{\partial x\partial \theta }-\frac{3}{4}\frac{\partial v}{\partial x}+\frac{1}{4R}\frac{\partial u}{\partial \theta })+{d}_{11}{(\frac{{\partial }^{2}w}{\partial {x}^{2}})}^{2}+\frac{{d}_{22}}{{R}^{4}}{(\frac{{\partial }^{2}w}{\partial {\theta }^{2}}-\frac{\partial v}{\partial \theta })}^{2}+\frac{2{d}_{12}}{{R}^{2}}(\frac{{\partial }^{2}w}{\partial {x}^{2}})(\frac{{\partial }^{2}w}{\partial {\theta }^{2}}-\frac{\partial v}{\partial \theta })+\frac{4{d}_{66}}{{R}^{2}}(\frac{{\partial }^{2}w}{\partial x\partial \theta }-\frac{3}{4}\frac{\partial v}{\partial x}+{\frac{1}{4R}\frac{\partial u}{\partial \theta })}^{2}]dxd\theta \mathrm{.}$$

Shell kinetic energy is symbolized by Ì and is stated as:

10 $${\rm{I}}=\frac{1}{2}{\int }_{0}^{L}{\int }_{0}^{2\pi }{\rho }_{t}[{(\frac{\partial u}{\partial t})}^{2}+{(\frac{\partial v}{\partial t})}^{2}+{(\frac{\partial w}{\partial t})}^{2}]\,Rd\theta d\mathrm{.}$$

Here variable *t* designates the time. Mass density is represented by *ρ* and *ρ*_*t*_ denotes the mass density for each unit length and it is expressed as:

11 $${\rho }_{t}={\int }_{-\frac{h}{2}}^{\frac{h}{2}}\rho dz\mathrm{.}$$

The Lagrange energy functional denoted by ${\mathcal L}$ for a cylinder-shaped shell is formulated by the difference of kinetic and strain energies as:

12 $$ {\mathcal L} =I-\Im .$$

## Numerical Procedure

The Rayleigh-Ritz procedure is used to achieve the natural frequencies of cylindrical shell. Now the displacement fields are presummed by the following relations:

13 $$\begin{array}{ccc}u(x,{\theta },t) & = & {x}_{m}U\,(x)\,\cos \,(n{\theta })\,sin{\boldsymbol{\omega }}t,\\ v(x,{\theta },t) & = & {y}_{m}V\,(x)\,\sin \,(n{\theta })\,cos{\boldsymbol{\omega }}t,\\ w(x,{\theta },t) & = & {z}_{m}W\,(x)\,\cos \,(n{\theta })\,sin{\boldsymbol{\omega }}t,\end{array}$$

where *x*_*m*_, *y*_*m*_ and *z*_*m*_ represent the amplitudes of vibration in the *x*, *θ* and *z* direction respectively, the axial and circumferential wave numbers of mode shapes are denoted by *m* and *n* respectively, *ω* signifies the angular vibration frequency of the shell wave. *U*(*x*), *V*(*x*), and *W*(*x*), denotes the axial model dependence in the longitudinal, circumferential and transverse directions respectively. Here we take $U(x)=\frac{d\phi (x)}{dx},\,V(x)=\phi (x),\,W(x)=\phi (x)$, where *φ*(*x*) represents the axial function which satisfies the geometric edge conditions.

The axial function *φ*(*x* is taken as the beam function in the following form,

14 $$\phi \,(x)={\beta }_{1}\cos \,h\,({\mu }_{m}x)+{\beta }_{2}cos\,({\mu }_{m}x)-{\sigma }_{m}\,({\beta }_{3}sinh\,({\mu }_{m}x)+{\beta }_{4}sin\,({\mu }_{m}x))$$

Here values of *β*_*i*_ are changed with respect to the edge conditions. (*i* = 1, 2, 3, 4) *μ*_*m*_ signify the roots of some transcendental equations and *σ*_*m*_ are parameters which depend on the values of *μ*_*m*_.

For generalization of this problem following non-dimensional parameters are used.

15 $$\begin{array}{rcl}\underline{{U}_{1}} & = & \frac{U(x)}{h},\,\underline{{V}_{1}}=\frac{V(x)}{h},\,\underline{{W}_{1}}=\frac{W(x)}{R},\\ \underline{{a}_{ij}} & = & \frac{{a}_{ij}}{h},\,\underline{{b}_{ij}}=\frac{{b}_{ij}}{{h}^{2}},\,\underline{{d}_{ij}}=\frac{{d}_{ij}}{{h}^{3}},\\ \alpha  & = & R/L,\,\beta =h/R,\,X=\frac{x}{L},\,\underline{{\rho }_{t}}=\frac{{\rho }_{t}}{h}.\end{array}$$

Now expression (13) is altered into the following form

16 $$\begin{array}{rcl}u(x,\theta ,t) & = & h{x}_{m}{U}_{1}\,\cos \,(n\theta )\,sin\omega t,\\ v(x,\theta, t) & = & h{y}_{m} {V}_{1}\,\sin \,(n\theta )\,cos\omega t,\\ w(x,\theta ,t) & = & R{z}_{m}{W}_{1}\,\cos \,(n\theta )\,sin\omega t\mathrm{.}\end{array}$$

After substituting expression (3.4) into the expressions for $\Im$ and Ì, we get $\Im$_*max*_, Ì_*max*_ and ${ {\mathcal L} }_{max}.$ Then Lagrangian functional ${ {\mathcal L} }_{max}$ transformed into the following form by applying the principle of maximum energy.

17 $${ {\mathcal L} }_{max}=\frac{\pi hLR}{2}[{R}^{2}{\omega }^{2}{\underline{\rho}}_{t}{\int }_{0}^{1}({\beta }^{2}{({x}_{m}\underline{{U}_{1}})}^{2}+{\beta }^{2}{({y}_{m}\underline{{V}_{1}})}^{2}+{({z}_{m}\underline{{W}_{1}})}^{2})dX-{\int }_{0}^{1}\{{\alpha }^{2}{\beta }^{2}\underline{{a}_{11}}{({x}_{m}\frac{d\underline{{U}_{1}}}{dX})}^{2}+\underline{{a}_{22}}{(-n\beta {y}_{m}\underline{{V}_{1}}+{z}_{m}\underline{{W}_{1}})}^{2}+2\alpha \beta \underline{{a}_{12}}\times ({x}_{m}\frac{d\underline{{U}_{1}}}{dX})(\,-\,n\beta {y}_{m}\underline{{V}_{1}}+{z}_{m}\underline{{W}_{1}})+\underline{{a}_{66}}{(\alpha \beta {y}_{m}\frac{d\underline{{V}_{1}}}{dX}+n\beta {x}_{m}\underline{{U}_{1}})}^{2}-2{\alpha }^{3}{\beta }^{2}\underline{{b}_{11}}({x}_{m}\frac{d\underline{{U}_{1}}}{dX})({{z}_{m}}^{2}\frac{{d}^{2}\underline{{W}_{1}}}{d{X}^{2}})-2\alpha {\beta }^{2}\underline{{b}_{12}}({x}_{m}\frac{d\underline{{U}_{1}}}{dX})\times (-{n}^{2}{z}_{m}\underline{{W}_{1}}+n\beta {y}_{m}\underline{{V}_{1}})-2{\alpha }^{2}\beta \underline{{b}_{12}}(\,-\,n\beta {y}_{m}\underline{{V}_{1}}+{z}_{m}\underline{{W}_{1}})({{z}_{m}}^{2}\frac{{d}^{2}\underline{{W}_{1}}}{d{X}^{2}})-2\beta \underline{{b}_{22}}(\,-\,n\beta {y}_{m}\underline{{V}_{1}}+{z}_{m}\underline{{W}_{1}})\,(\,-\,{n}^{2}{z}_{m}\underline{{W}_{1}}+n\beta {y}_{m}\underline{{V}_{1}})-4\beta \underline{{b}_{66}}(\alpha \beta {y}_{m}\frac{d\underline{{V}_{1}}}{dX}+n\beta {x}_{m}\underline{{U}_{1}})(n\alpha {z}_{m}\frac{d\underline{{W}_{1}}}{dX}-\frac{3\alpha \beta {y}_{m}}{4}\frac{d\underline{{V}_{1}}}{dX}+\frac{n\beta }{4}{x}_{m}\underline{{U}_{1}})+{\alpha }^{4}{\beta }^{2}\underline{{d}_{11}}{({{z}_{m}}^{2}\frac{{d}^{2}\underline{{W}_{1}}}{d{X}^{2}})}^{2}+{\beta }^{2}\underline{{d}_{22}}{(-{n}^{2}{z}_{m}\underline{{W}_{1}}+n\beta {y}_{m}\underline{{V}_{1}})}^{2}+2{\alpha }^{2}{\beta }^{2}\underline{{d}_{12}}({{z}_{m}}^{2}\frac{{d}^{2}\underline{{W}_{1}}}{d{X}^{2}})(\,-\,{n}^{2}{z}_{m}\underline{{W}_{1}}+n\beta {y}_{m}\underline{{V}_{1}})+\,4\underline{{d}_{66}}{(n\alpha {z}_{m}\frac{d\underline{{W}_{1}}}{dX}-\frac{3\alpha \beta {y}_{m}}{4}\frac{d\underline{{V}_{1}}}{dX}+\frac{n\beta }{4}{x}_{m}\underline{{U}_{1}})}^{2}\}dX]\mathrm{.}$$

Rayleigh-Ritz procedure is employed to get the eigenvalue form problem of the shell frequency equation. The Lagrangian energy functional ${ {\mathcal L} }_{{\max }}$ is minimized with regarding the vibration amplitudes *x*_*m*_, *y*_*m*_ and *z*_*m*_ as follows,

18 $$\frac{\partial { {\mathcal L} }_{{\max }}}{\partial {x}_{m}}=\frac{\partial { {\mathcal L} }_{{\max }}}{\partial {y}_{m}}=\frac{\partial { {\mathcal L} }_{{\max }}}{\partial {z}_{m}}=0.$$

The obtained equations by arrangements of terms are written in matrix form as

19 $$\{[C]-{{\rm{\Omega }}}^{2}[{,}\kern -.5em \rm{M}]\}\underline{X}=0$$

where

20 $${{\rm{\Omega }}}^{{\rm{2}}}={R}^{2}{\omega }^{2}{\underline{\rho }}_{t},$$

where [C] and [${,}\kern-.5em \rm{M}$] are the stiffness and mass matrices of the cylindrical shell respectively and its values are given supplementary file, and [C] contains the terms related material moduli nd the mass matrix [${,}\kern -.5em \rm{M}$] contains terms associated with shell mass,

21 $$\mathop{\underline{X}}\limits^{{^{\prime} }}=[{x}_{m},{y}_{m},{z}_{m}],$$

the shell vibrations are determined after solving the eigenvalue equation (19) with the help of MATLAB software.

## Classifications of Materials

In present study a cylindrical shell is considered constructed from three layers, the internal and external layers are fabricated by isotropic material while the central layer is constructed from FG materials nickel and stainless steel. The volume fractions^14^ of the shell middle layer constructed from two constituents using trigonometric volume fraction law (VFL) are given by the following relations:

22 $${V}_{f1}=si{n}^{2}({[\frac{3z}{h}+\frac{1}{2}]}^{\upsilon }),\,\,{V}_{f2}=co{s}^{2}({[\frac{3z}{h}+\frac{1}{2}]}^{\upsilon })\,\,\,\,\,0\le \upsilon \le \infty \mathrm{.}$$

These relations satisfy the VFL i.e.*V*_*f*1_+*V*_*f*2_ = 1, where *h* is the shell thickness and *υ* denotes the power law exponent. It is presumed that each layer is of thickness *h*/3. Following are the material parameters: ${E}_{1},{{{\rm{{-}\kern-0.5em \lambda}}}}_{1},{\rho }_{1}\,and\,{E}_{2},{\rho }_{2},{{{\rm{{-}\kern-0.5em \lambda}}}}_{2}$ for nickel and stainless steel respectively. Then the effective material quantities: ${E}_{fgm},{{{\rm{{-}\kern-0.5em \lambda}}}}_{1fgm}\,and\,{\rho }_{fgm}$ for one type of the configuration are given as:

23 $$\begin{array}{rcl}{E}_{fgm} & = & [{E}_{1}-{E}_{2}]\,{si}{{n}}^{2}({[\frac{3z}{h}+\frac{1}{2}]}^{\upsilon })+{E}_{2},\\ {{{\rm{{-}\kern-0.5em \lambda}}}}_{fgm} & = & [{{{\rm{{-}\kern-0.5em \lambda}}}}_{1}-{{{\rm{{-}\kern-0.5em \lambda}}}}_{2}]\,{si}{{n}}^{2}({[\frac{3z}{h}+\frac{1}{2}]}^{\upsilon })+{{{\rm{{-}\kern-0.5em \lambda}}}}_{2},\\ {\rho }_{fgm} & = & [{\rho }_{1}-{\rho }_{2}]\,{si}{{n}}^{2}({[\frac{3z}{h}+\frac{1}{2}]}^{\upsilon })+{\rho }_{2.}\end{array}$$

From expression (23) at *z* = *−h/6*, *E*_*fgm*_ = *E*_2_, ${{{\rm{{-}\kern-0.5em \lambda}}}}_{fgm}={{{\rm{{-}\kern-0.5em \lambda}}}}_{2}$, *ρ*_*fgm*_ = *ρ*_2_ and the material properties at *z* = *h/6* becomes:

$$\begin{array}{rcl}{E}_{fgm} & = & [{E}_{1}-{E}_{2}]\,{si}{{n}}^{2}1+{E}_{2}\\ {{{\rm{{-}\kern-0.5em \lambda}}}}_{fgm} & = & [{V}_{1}-{V}_{2}]\,{si}{{n}}^{2}1+{{{\rm{{-}\kern-0.5em \lambda}}}}_{2},\\ {\rho }_{fgm} & = & [{\rho }_{1}-{\rho }_{2}]\,{si}{{n}}^{2}1+{\rho }_{2}.\end{array}$$

Thus the shell is consisted of purely stainless steel at *z* = *−h/6* and the properties of material are combination of stainless steel and nickel at *z* = +*h/6*. The stiffness moduli are modified as:

$$\begin{array}{rcl}{a}_{ij} & = & {a}_{ij}(iso)+{a}_{ij}(FGM)+{a}_{ij}(iso),\\ {b}_{ij} & = & {b}_{ij}(iso)+{b}_{ij}(FGM)+{b}_{ij}(iso),\\ {d}_{ij} & = & {d}_{ij}(iso)+{d}_{ij}(FGM)+{d}_{ij}(iso),\end{array}$$

where *i* = 1, 2, 6 and *(iso)* represents the internal and external isotropic layers and FGM represents the central functionally graded material layer.

## Results and Discussion

Results for an isotropic cylindrical shell with following edge conditions, simply supported-simply supported (${{,} \kern -.3em \rm{s}}$-${{,} \kern -.3em \rm{s}}$), clamped-clamped (*ς*- *ς*) and clamped-free (*ς*-${{,} \kern -.27em \rm{f}}$), are compared with the results available in open literature to ensure the validity, authenticity and robustness of the current technique. Tables 1 and 2 show the comparisons of frequency parameters with those in the Zhang *et al*.^7^ for ${{,} \kern -.3em \rm{s}}$-${{,} \kern -.3em \rm{s}}$ and *ς*- *ς* isotropic cylindrical shells. Comparison of natural frequencies (Hz) with those available in Loy & Lam^4^ for *ς*-${{,} \kern -.27em \rm{f}}$ isotropic cylindrical shell is presented in the Table 3. It can be noticed clearly that the current results are in agreement with the results in open literature.

**Table 1:** Comparison of frequency parameter ${\rm{\Delta }}=\omega R\sqrt{(1-{{{\rm{{-}\kern-0.5em \lambda}}}}^{2})\rho /E}$ of ${{,} \kern -.3em \rm{s}}$-${{,} \kern -.3em \rm{s}}$ shell.

	*υ*			
	1	2	3	4
Zhang *et al*.^7^	0.0161	0.03927	0.10981	0.21028
Present	0.0161	0.03927	0.10981	0.21028
Difference%	0.006	0.001	0.001	0.0000

(*m* = 1, $${{\rm{{-}\kern-0.5em \lambda}}}$$ = 0.3, *L* = 20, *h* = 0.05, *R* = 1).

**Table 2:** Comparison of frequency parameter ${\rm{\Delta }}=\omega R\sqrt{(1-{{{\rm{{-}\kern-0.5em \lambda}}}}^{2})\rho /E}$ of *ς*- *ς* shell.

	*υ*			
	1	2	3	4
Zhang *et al*.^7^	0.03285	0.04064	0.10997	0.21032
Present	0.0344	0.04077	0.11001	0.21038
Difference%	4.7	0.33	0.03	0.02

(*m* = 1, $${{\rm{{-}\kern-0.5em \lambda}}}$$ = 0.3, *L* = 20, *h* = 0.05, *R* = 1).

**Table 3:** Comparison of frequency parameter ${\rm{\Delta }}=\omega R\sqrt{(1-{{{\rm{{-}\kern-0.5em \lambda}}}}^{2})\rho /E}$ of *ς*-${{,} \kern -.27em \rm{f}}$ cylindrical shell.

	*υ*				
	2	3	4	5	6
Loy & Lam^4^	319.5	769.9	1465.7	2366.9	3479
Present	319.52	769.86	1465.73	2366.93	3470

(*m* = 1, ${{\rm{{-}\kern-0.5em \lambda}}}$ = 0.28, *h* = 63.5 mm. *R* = 1.63 mm. *L* = 502 mm).

Table 4 represents the types of three layered FGM cylinder shaped shell by interchanging the FG constituent materials. where *Z*_1_, *Z*_2_ and *Z*_3_ represent Aluminium, Stainless Steel and Nickel respectively. Material properties for the above materials are presented in refs^5,19^. Different arrangements of thickness for shell layers are presented in Table 5.

**Table 4:** Configurations of shell types.

Types of Shell	Internal Isotropic Layer	Central FGM Layer	External Isotropic Layer
Type I	*Z*_1_	*Z*_2_/*Z*_3_	*Z*_1_
Type II	*Z*_1_	*Z*_3_/*Z*_2_	*Z*_1_

**Table 5:** Thickness differences of shell layers.

Thickness arrangements	Internal Layer	Central Layer	External Layer
Case-I	*q*_1_	*q*_1_	*q*_1_
Case-II	*q*_2_	*q*_3_	*q*_2_
Case-III	*q*_4_	*q*_5_	*q*_4_

Here *q*_1_ = *h*/3, *q*_2_ = *h*/4, *q*_3_ = *h*/2, *q*_4_ = *h*/5, *q*_5_ = 3*h*/5.

Tables 6 and 7 represent natural frequencies (Hz) functionally graded material cylindrical shell versus against *n* for case-II, type-I & II with different power exponent law *γ* respectively. In these tables influence of *υ* is examined which is different for both types. The natural frequencies (Hz) are decreased for type-I and increased for type-II less than 1% when power exponent law increased from *υ* = 1–20 for *n* = 1–5. Hence natural frequencies are affected by the configuration of the essential materials in the three layered CS.

**Table 6:** Variation of NFs (Hz) for various power exponent law *υ* against *n* for shell type-I ((*m* = 1, *L/R* = 50, *h/R* = 0.001).

*n*	*υ* = 1	*υ* = 2	*υ* = 3	*υ* = 5	*υ* = 10	*υ* = 15	*υ* = 20
1	1.6067	1.6024	1.6005	1.5988	1.5973	1.5968	1.5965
2	0.8251	0.8229	0.8220	0.8212	0.8205	0.8202	0.8201
3	1.8493	1.8446	1.8426	1.8408	1.8393	1.8388	1.8385
4	3.5182	3.5091	3.5053	3.5019	3.4991	3.4981	3.4976
5	5.6857	5.6711	5.6650	5.6594	5.6549	5.6533	5.6524

**Table 7:** Variation of natural frequencies for various power exponent law *υ* against *n* for shell type-II (*m* = 1, *L* = 50, *h* = 0.001, *R* = 1).

*n*	*υ* = 1	*υ* = 2	*υ* = 3	*υ* = 5	*υ* = 10	*υ* = 15	*υ* = 20
1	1.6245	1.629	1.631	1.6328	1.6343	1.6349	1.6352
2	0.8338	0.836	0.837	0.8379	0.8386	0.8389	0.839
3	1.8681	1.8731	1.8752	1.8771	1.8786	1.8792	1.8795
4	3.5539	3.5633	3.5672	3.5709	3.5739	3.5749	3.5755
5	5.7434	5.7586	5.765	5.7709	5.7757	5.7774	5.7783

**Figure 2:**
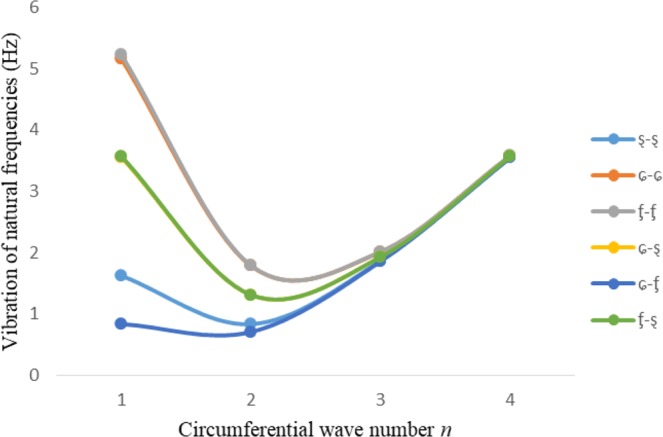
Vibrations of natural frequencies (Hz) for case-I Type I cylindrical shell against (n).

**Figure 3:**
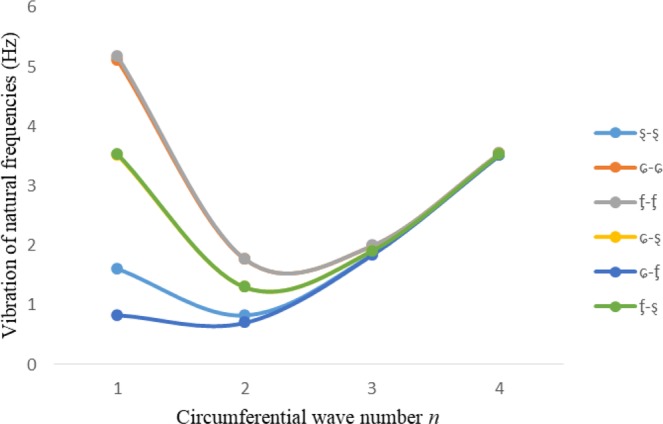
Vibrations of natural frequencies (Hz) for case-II Type I cylindrical shell against (n).

**Figure 4:**
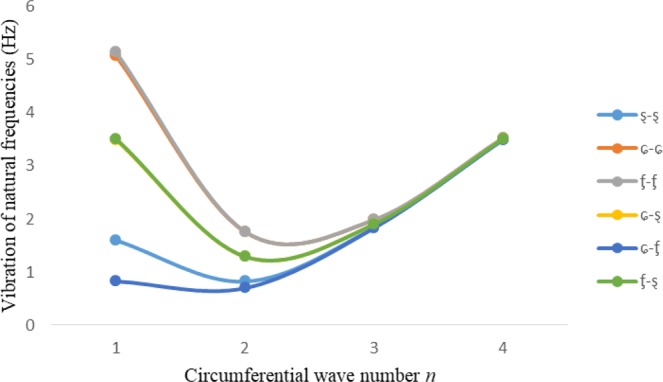
Vibrations of natural frequencies (Hz) for case-III Type I cylindrical shell against (n).

**Figure 5:**
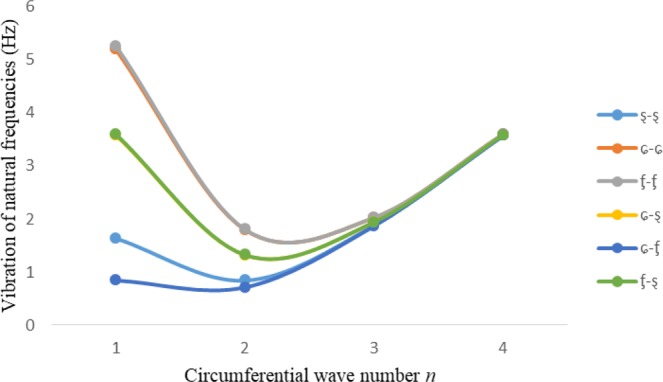
Vibrations of natural frequencies (Hz) for case-I Type II cylindrical shell against (n).

**Figure 6:**
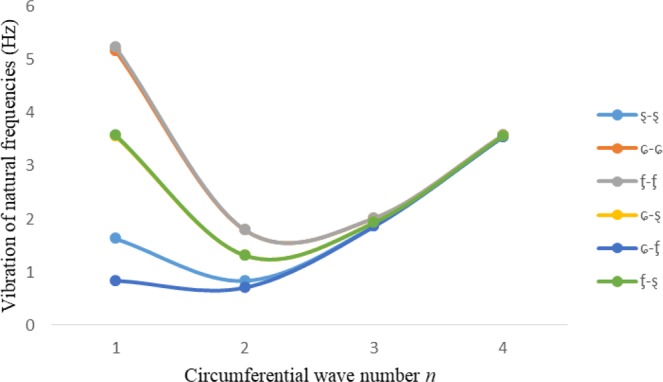
Vibrations of natural frequencies (Hz) for case-II Type II cylindrical shell against (n).

**Figure 7:**
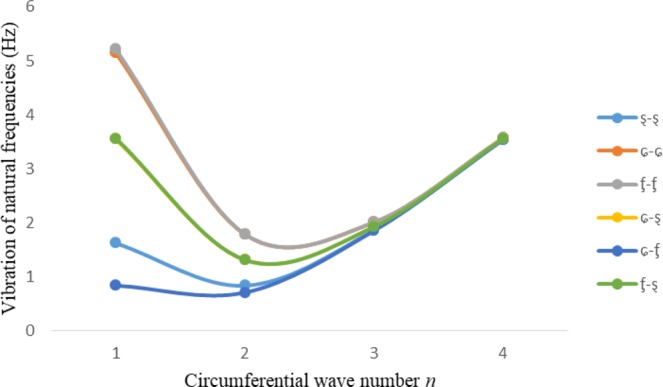
Vibrations of natural frequencies (Hz) for case-III Type II cylindrical shell against (n).

Figures 2–7 represent the natural frequencies (NFs) (Hz) of FGM cylinder-shaped shell against n for different thickness of the central layer under six edge conditions; ${{,} \kern -.3em \rm{s}}$-${{,} \kern -.3em \rm{s}}$, *ς*-*ς*, ${{,} \kern -.27em \rm{f}}$-${{,} \kern -.27em \rm{f}}$, *ς*-${{,} \kern -.3em \rm{s}}$ (clamped-simply supported), *ς*-${{,} \kern -.27em \rm{f}}$ (clamped-free), ${{,} \kern -.27em \rm{f}}$-${{,} \kern -.3em \rm{s}}$ (free-simply supported). In Figs 2–4 Natural frequencies are presented for cylindrical shells of type I. Natural frequencies decrease for n = 2 and starts increase at n = 3 in each case. It is seen that the natural frequencies are minimum for clamped-free edge condition as compare to other five edge conditions and its maximum for free-free end point condition. The behavior of natural frequencies (Hz) remains same for all cases. Natural frequencies decreased <1% when thickness of the shell middle layer increased 66% or 100%. Figures 5–7 demonstrate the results for cylindrical shells of type-II. It is clearly seen that the natural frequencies are little high for cylindrical shells of type-II as compare to type-I shells.

**Figure 8:**
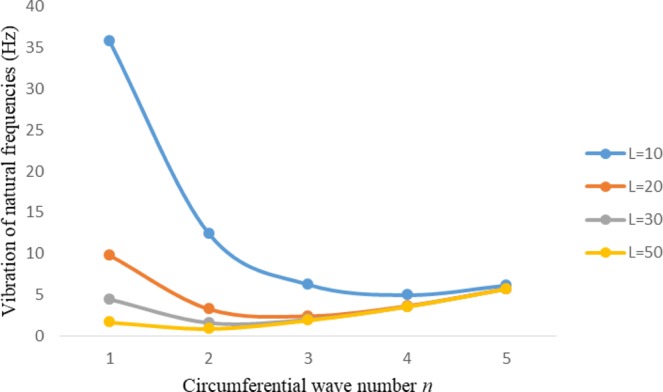
Vibration of natural frequencies (Hz) for length to radius ratios against n for FGM shell of Case-II with ${{,} \kern -.3em \rm{s}}$-${{,} \kern -.3em \rm{s}}$ edge conditions.

**Figure 9:**
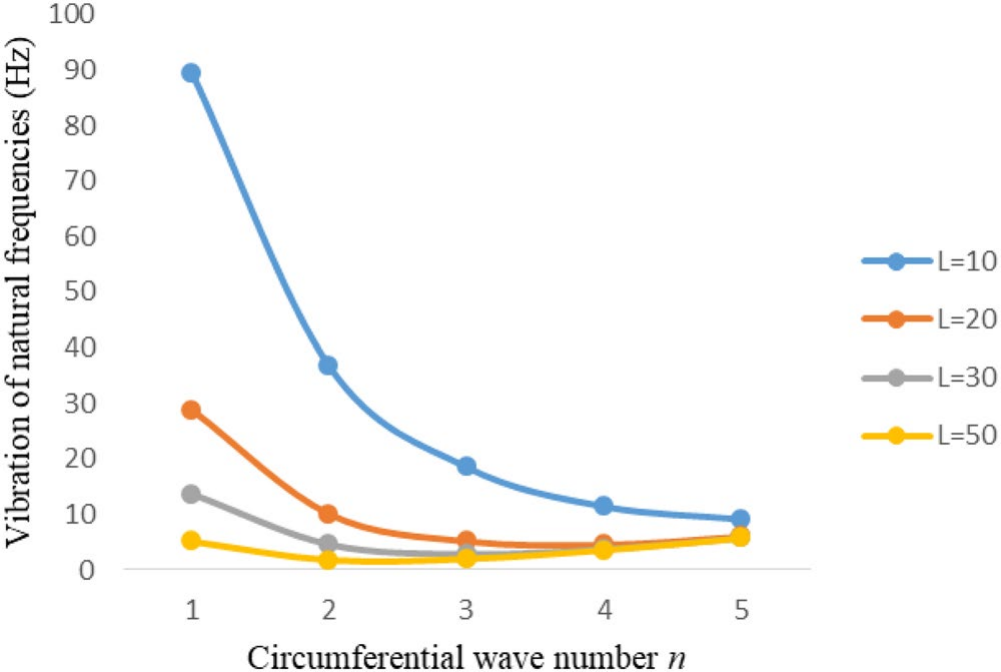
8 Vibration of natural frequencies (Hz) for length to radius ratios against n for FGM shell of Case-II with *ς* -*ς* edge conditions.

**Figure 10:**
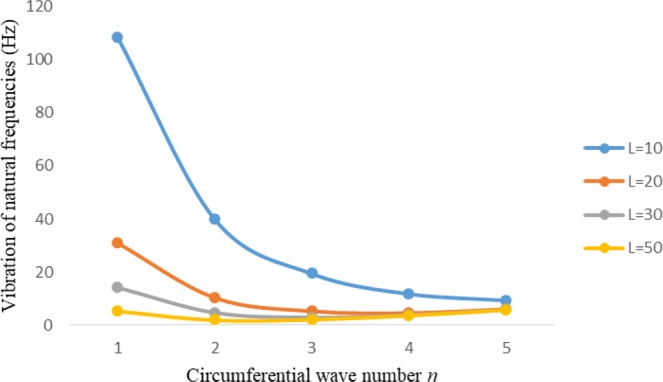
9 Vibration of natural frequencies (Hz) for length to radius ratios against n for FGM shell of Case-II with ${{,} \kern -.27em \rm{f}}$-${{,} \kern -.27em \rm{f}}$ edge conditions.

**Figure 11:**
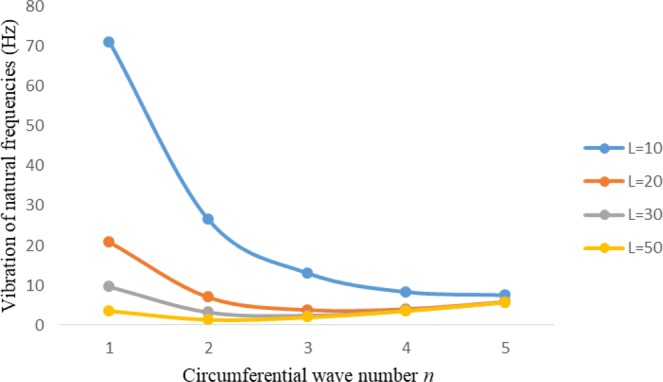
Vibration of natural frequencies (Hz) for length to radius ratios against n for FGM shell of Case-II with *ς*-${{,} \kern -.3em \rm{s}}$.

**Figure 12:**
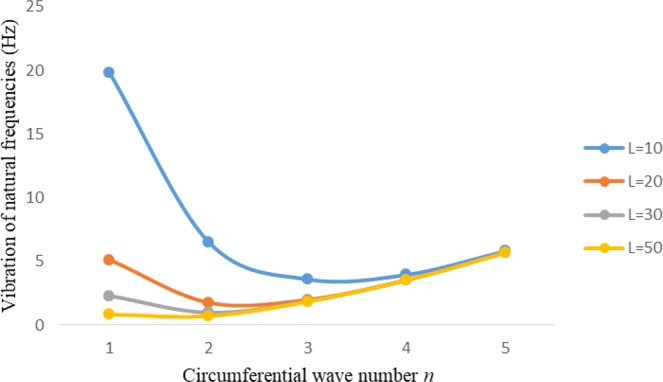
Vibration of natural frequencies (Hz) for length to radius ratios against n for FGM shell of Case-II with *ς* -${{,} \kern -.27em \rm{f}}$ edge conditions.

**Figure 13:**
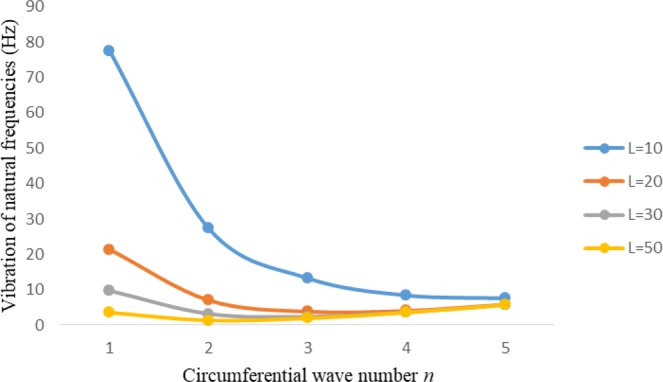
Vibration of natural frequencies (Hz) for length to radius ratios against n for FGM shell of Case-II with ${{,} \kern -.27em \rm{f}}$-${{,} \kern -.3em \rm{s}}$ edge conditions.

**Figure 14:**
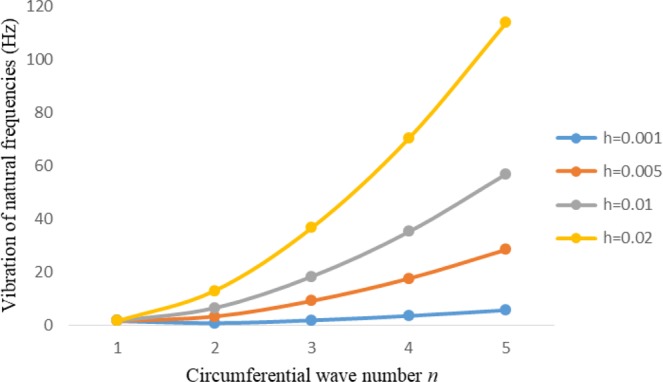
Vibration of natural frequencies for thickness to radius ratios against n for FGM shell of case-II with ${{,} \kern -.3em \rm{s}}$-${{,} \kern -.3em \rm{s}}$ edge conditions.

**Figure 15:**
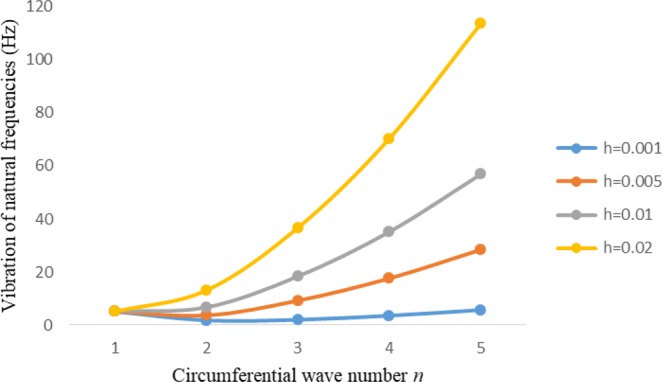
Vibration of natural frequencies for thickness to radius ratios against n for FGM shell of case-II with *ς*-*ς* edge conditions.

**Figure 16:**
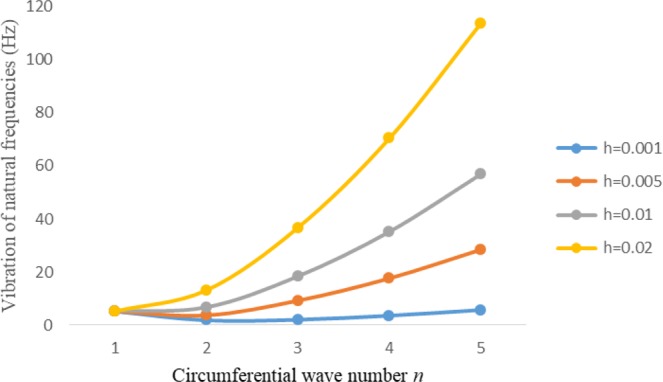
Vibration of natural frequencies for thickness to radius ratios against n for FGM shell of case-II with ${{,} \kern -.27em \rm{f}}$-${{,} \kern -.27em \rm{f}}$ edge conditions.

**Figure 17:**
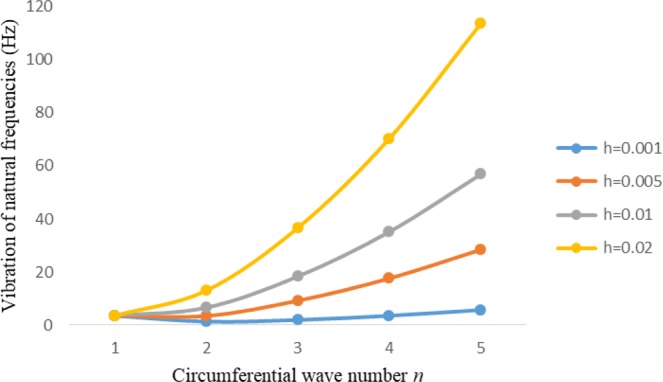
Vibration of natural frequencies for thickness to radius ratios against n for FGM shell of case-II with *ς* -${{,} \kern -.3em \rm{s}}$ edge conditions.

**Figure 18:**
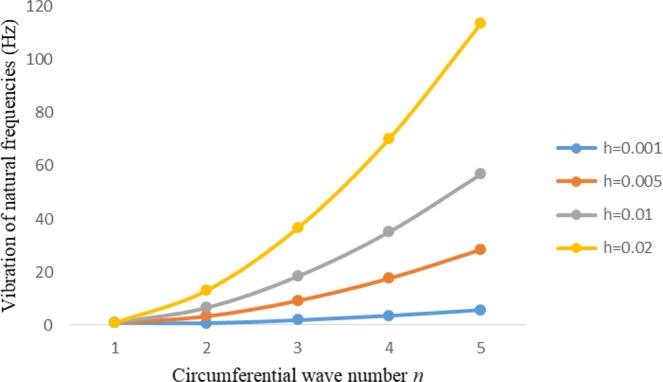
Vibration of natural frequencies for thickness to radius ratios against n for FGM shell of case-II with *ς*-${{,} \kern -.27em \rm{f}}$ edge conditions.

**Figure 19:**
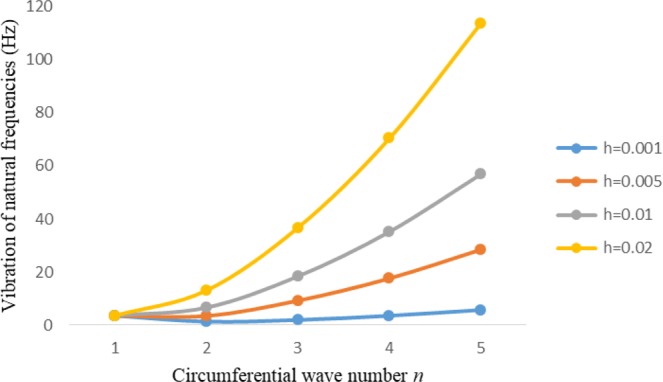
Vibration of natural frequencies for thickness to radius ratios against n for FGM shell of case-II with ${{,} \kern -.27em \rm{f}}$-${{,} \kern -.3em \rm{s}}$ edge conditions.

Figures 8–13 show the behavior of natural frequencies (Hz) versus n for various L/R ratios and for various edge conditions. It is seen that the natural frequencies (Hz) are decreased when the L/R ratios are increased. When L/R ratios are increased from 10 to 20, 30, and 50 then natural frequencies are decreased 72%, 87% and 95% respectively for n = 1. Natural frequencies (Hz) for different h/R ratios against n are presented in Figs 14–19 under six edge conditions. Natural frequencies (Hz) are increased with the increasing h/R ratios. In these figures, frequencies first decreased from n = 1 to 2 then increased from 2 to onwards. Natural frequencies are increased with the increasing h/R ratios from 0.001 to 0.005, 0.005 to 0.01 and 0.01 to 0.02 at n = 2 for different boundary conditions such as for simply supported - simply supported boundary condition 298%, 98% and 100% for *ς*-*ς* and f-f boundary conditions 105%, 84% and 95% for *ς*-${{,} \kern -.3em \rm{s}}$ and ${{,} \kern -.27em \rm{f}}$-${{,} \kern -.3em \rm{s}}$ boundary condition 165%, 92% and 98% for *ς*-${{,} \kern -.27em \rm{f}}$ boundary condition 365%, 100% and 100% for respective ratios. Thus Natural frequencies affected significantly by h/R ratios.

## Conclusions

In present study, frequency analysis of three layered FGM cylinder shaped shell is done for different thickness of the shell middle layer. Strain and curvature displacement relationships are adopted from Sander’s theory. To solve the current problem Rayleigh Ritz method is employed. Natural frequencies are examined for six edge conditions. It is noticed that Natural frequencies becomes minimum with the increase in thickness of the shell FGM middle layer. These also decreased with the increased of *L/R* ratios. When *L/R* ratios increased 100%, 200% and 500% then natural frequencies decreased 72%, 87% and 95% respectively for *n* = 1. Frequencies increased with the increased of *h/R* ratios. Thickness to radius ratios has significant effect on natural frequencies (Hz).

## Supplementary information

Appendix
