# Supplementary material for: Vibration frequency analysis of three-layered cylinder shaped shell with effect of FGM central layer thickness
Source: Sci Rep. 2019 Feb 7;9:1566. doi: 10.1038/s41598-018-38122-0 (PMC6367515; doi:10.1038/s41598-018-38122-0)
Supplement: Supplementary file 1 — Appendix [file 41598_2018_38122_MOESM1_ESM.pdf]

Vibration frequency analysis of three-layered cylinder shaped shell with effect of FGM central layer thickness

Madiha Ghamkhar[1], Muhammad Nawaz Naeem[1], Muhammad Imran[1,*], Muhammad Kamran[2,*], and Constantinos Soutis[3]

[1]Department of Mathematics, Government College University Faisalabad, Pakistan
[2]Department of Mathematics, COMSATS University Islamabad, Wah Campus, Pakistan
[3]Aerospace Research Institute and Northwest Composites Centre, The University of Manchester, UK
[*]Corresponding Author: drmimranchaudhry@gmail.com

Appendix

$$C_{11} = \alpha^2\beta^2\underline{a_{11}}I_1 + n^2\beta^2(\underline{a_{66}} - \beta\underline{b_{66}} + \frac{\beta^2\underline{d_{66}}}{4})I_2,$$

$$C_{12} = -n\alpha\beta^2\left(\underline{a_{12}} + \beta\underline{b_{12}}\right)I_3 + n\alpha\beta^2(\underline{a_{66}} + \beta\underline{b_{66}} - \frac{3\beta^2\underline{d_{66}}}{4})I_4,$$

$$C_{13} = \alpha\beta\left(\underline{a_{12}} + n^2\beta\underline{b_{12}}\right)I_5 + n^2\alpha\beta^2\left(-2\underline{b_{66}} + \beta\underline{d_{66}}\right)I_6 - \alpha^3\beta^2\ \underline{b_{11}}I_7,$$

$$C_{21} = C_{12}$$

$$C_{22} = n^2\beta^2\left(\underline{a_{22}} + 2\beta\underline{b_{22}} + \beta^2\underline{d_{22}}\right)I_8 + \alpha^2\beta^2(\underline{a_{66}} + 3\beta\underline{b_{66}} + 9\beta^2\underline{d_{66}})I_9,$$

$$C_{23} = -n\beta\left(\underline{a_{22}} + \left(n^2+1\right)\beta\underline{b_{22}} + n^2\beta^2\underline{d_{22}}\right)I_{10} - n\alpha^2\beta^2(2\underline{b_{66}},$$

$$+3\beta\underline{d_{66}}I_{11} + n\alpha^2\beta^2(\underline{b_{12}} + \beta\underline{d_{12}})I_{12},$$

$$C_{31} = C_{13},$$

$$C_{32} = C_{23},$$

$$C_{33} = \left(\underline{a_{22}} + 2n^2\beta\underline{b_{22}} + n^4\beta^2\underline{d_{22}}\right)I_{13} + 4n^2\alpha^2\beta^2\underline{d_{66}}I_{14},$$

$$-\alpha^2\beta\left(\underline{b_{12}} + n^2\beta\underline{d_{12}}\right)I_{15} + \alpha^4\beta^2\underline{d_{11}}I_{16},$$

where

$$I_1 = \int_0^L \left(d\underline{U_1}/dX\right)^2 dX,\ I_2 = \int_0^L \underline{U_1}^2 dX,\ I_3 = \int_0^L \left(d\underline{U_1}/dX\right)\underline{V_1}dX,$$

$$I_4 = \int_0^L \left(d\underline{V_1}/dX\right)\underline{U_1}dX,$$

$$I_6 = \int_0^L \left(d\underline{W_1}/dX\right)\underline{U_1}dX,\ \ I_7 = \int_0^L \left(d\underline{U_1}/dX\right)\left(d^2\underline{W_1}/dX^2\right)dX,$$

$$I_8 = \int_0^L \underline{V_1}^2 dX,\ \ I_9 = \int_0^L \left(d\underline{V_1}/dX\right)^2\underline{U_1}dX,\ \ I_{10} = \int_0^L \underline{V_1 W_1}dX,$$

$$I_{11} = \int_0^L \left(d\underline{V_1}/dX\right)\left(d\dddot{\underline{V_1}}/dX\right)_1 dX,\, I_{12} = \int_0^L \left(d^2\underline{W_1}/dX^2\right)\underline{V_1}dX,$$

$$I_{13} = \int_0^L \underline{W_1}^2 dX,\, I_{14} = \int_0^L \left(d\underline{W_1}/dX\right)^2 dX,$$

$$I_{15} = \int_0^L 2\left(d^2\underline{W_1}/dX^2\right)\underline{W_1}dX,\;\; I_{16} = \int_0^L \left(d^2\underline{W_1}/dX^2\right)^2 dX,$$

and

$$\underline{a_{11}} = \frac{2E}{3(1-\lambda^2)} + \frac{1}{(1-\lambda_1{}^2)}\left[\frac{E_1-E_2}{3(\upsilon+1)} + \frac{E_2}{3}\right],$$

$$\underline{a_{12}} = \frac{2E\lambda}{3(1-\lambda^2)} + \frac{\lambda_1}{(1-\lambda_1{}^2)}\left[\frac{E_1-E_2}{3(\upsilon+1)} + \frac{E_2}{3}\right],$$

$$\underline{a_{66}} = \frac{E}{3(1+\lambda)} + \frac{1}{2(1+\lambda_1)}\left[\frac{E_1-E_2}{3(\upsilon+1)} + \frac{E_2}{3}\right],$$

$$\underline{a_{22}} = \underline{a_{11}},$$

$$\underline{b_{11}} = \frac{E_1-E_2}{\left(1-\lambda_1{}^2\right)}\left[\frac{1}{18(\upsilon+1)} + \frac{1}{9(\upsilon+1)(\upsilon+2)}\right],$$

$$\underline{b_{12}} = \frac{\lambda_1(E_1-E_2)}{\left(1-\lambda_1{}^2\right)}\left[\frac{1}{18(\upsilon+1)} + \frac{1}{9(\upsilon+1)(\upsilon+2)}\right],$$

$$\underline{b_{66}} = \frac{E_1-E_2}{2(1+\lambda_1)}\left[\frac{1}{18(\upsilon+1)} + \frac{1}{9(\upsilon+1)(\upsilon+2)}\right],$$

$$\underline{b_{22}} = \underline{b_{11}},$$

$$\underline{d_{11}} = \frac{13E}{162(1-\lambda^2)} + \frac{E_1-E_2}{\left(1-\lambda_1{}^2\right)}\left[\frac{1}{108(\upsilon+1)} - \frac{1}{27(\upsilon+1)(\upsilon+2)}\right.$$

$$\left. - \frac{2}{27(\upsilon+1)(\upsilon+2)(\upsilon+3)}\right] + \frac{E_2}{324\left(1-\lambda_1{}^2\right)}$$

$$\underline{d_{12}} = \frac{13E\lambda}{162(1-\lambda^2)} + \frac{\lambda_1(E_1-E_2)}{\left(1-\lambda_1{}^2\right)}\left[\frac{1}{108(\upsilon+1)} - \frac{1}{27(\upsilon+1)(\upsilon+2)}\right.$$

$$\left. - \frac{2}{27(\upsilon+1)(\upsilon+2)(\upsilon+3)}\right] + \frac{E_2}{324\left(1-\lambda_1{}^2\right)}$$

$$\underline{d_{66}} = \frac{13E}{324(1+\lambda)} + \frac{E_1-E_2}{2(1+\lambda_1)}\left[\frac{1}{108(\upsilon+1)} - \frac{1}{27(\upsilon+1)(\upsilon+2)}\right.$$

$$\left. - \frac{2}{27(\upsilon+1)(\upsilon+2)(\upsilon+3)}\right] + \frac{E_2}{648(1+\lambda_1)}$$

$$\underline{d_{22}} = \underline{d_{11}}$$

and

$$M_{11} = \beta^2 I_2,\; M_{22} = \beta^2 I_8,\; M_{33} = I_{13},$$

$$M_{12} = M_{13} = M_{21} = M_{23} = M_{31} = M_{32} = 0.$$
